# Unveiling the unprecedented – Very late brain metastasis in cholangiocarcinoma: A case report and comprehensive analysis

**DOI:** 10.1097/MD.0000000000039130

**Published:** 2024-07-26

**Authors:** Seungah Cha, Chang-Hoon Lee, Kyu Yun Jang, So-Yeon Jeon

**Affiliations:** a Division of Hematology/Oncology, Department of Internal Medicine, Jeonbuk National University Hospital, Jeonju, Republic of Korea; b Department of Pathology, Jeonbuk National University Hospital, Jeonju, Republic of Korea.

**Keywords:** brain metastasis, case report, cholangiocarcinoma, late recurrence

## Abstract

**Rationale:**

Cholangiocarcinoma (CCA) frequently invades nearby lymph nodes, the liver, and lungs. The liver and lungs are also common anatomic sites for the first recurrence of CCA. However, metastasis to the brain is exceptionally rare.

**Patient concerns:**

A 79-year-old male patient who was diagnosed with distal CCA and underwent pylorus-preserving pancreaticoduodenectomy along with adjuvant chemotherapy 13-years ago visited the neurosurgery outpatient department. He complained of dysarthria and right leg weakness that had started 7 days previously.

**Diagnoses:**

Brain computed tomography (CT) showed a 32 mm × 28 mm mass in the left frontal lobe with peripheral ring enhancement and vasogenic edema. A tumor mass removal operation was performed, and pathological examination revealed metastatic adenocarcinoma. Immunohistochemistry analysis revealed negativity for thyroid transcription factor-1 and napsin A, and positivity for cytokeratin (CK)7, CK20, and CK19. Simultaneously, Chest CT, abdomen–pelvis CT and 18-Fluoro-deoxyglucose positron emission tomography showed only two small nodules in the left upper lung, with no evidence of locoregional recurrence in the abdominal cavity. Considering these CT, positron emission tomography-CT, and pathologic findings, very late recurrence of biliary tract cancer with brain and lung metastases was suggested.

**Interventions and outcomes:**

A therapeutic plan involving systemic chemotherapy with gemcitabine and cisplatin was proposed, but the patient refused further chemotherapy.

**Lessons:**

This case highlights the unpredictable nature of metastatic patterns in CCA, where brain metastasis occurs very late, preceding locoregional recurrence in the liver. This challenges conventional expectations and underscores the need for vigilant surveillance and consideration of atypical metastatic sites in long-term survivors of CCA.

## 1. Introduction

Cholangiocarcinoma (CCA), the second most common primary liver cancer, originates from the hepatic biliary system and is categorized into 3 subtypes based on its site of origin: intrahepatic CCA (iCCA), perihilar CCA, and distal hepatic CCA.^[1]^ The latter 2 subtypes, collectively referred to as extrahepatic CCA, comprise approximately 40 % of all CCAs.^[2]^

Notably, CCA frequently invades neighboring lymph nodes, the liver, and the lungs, with the liver and lungs being common anatomic sites for its first recurrence. Conversely, metastasis to distant sites, such as the bone, is infrequent, and brain metastasis is exceptionally rare as a recurrent site.^[3]^ Given that complete surgical resection with negative margins represents the only potentially curative treatment for CCA, patients presenting with distant metastasis ultimately have a discouraging prognosis.

Particularly challenging is the limited penetration of systemic chemotherapy into the brain, compounded by the limited availability of clinical trials addressing brain metastasis from biliary tract cancer (BTC). These constraints significantly restrict the treatment options available for patients with CCA. Frega et al reported that a median overall survival (OS) of 3.7 months (ranging from 0.9 to 17.8) following the identification of brain metastasis and 23 months (ranging from 9.9 to 5 7.6) from the initial diagnosis of CCA. The median duration between the diagnosis of cancer and the identification of brain metastases was 13.6 months (ranging from 7.3 to 52.8).^[4]^

Here, we present an unusual case of a 79-year-old male with a remarkable history of distal CCA who presented with neurological symptoms indicative of brain metastasis. The rarity of such occurrences emphasizes the complexity of CCA metastatic behavior and the need for careful monitoring in long-term survivors.

## 2. Case description

A 79-year-old male patient with a history of distal CCA visited the neurosurgery outpatient department complaining of dysarthria and right leg weakness that had started 7 days ago. His medical history traces back to 13-years ago when he was diagnosed with distal CCA, pathologic stage T4N0M0. He underwent curative resection with pylorus-preserving pancreaticoduodenectomy and adjuvant chemotherapy with oral tegafur/uracil, resulting in complete clinical and radiologic responses.

**Figure 1.**
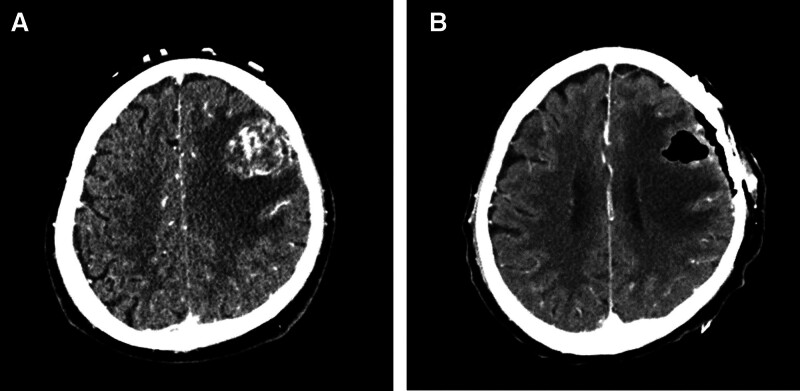
Enhanced brain CT. (A) An enhanced brain computed tomography (CT) scan showed a rim-enhancing mass with vasogenic edema in the left frontal lobe. (B) Follow-up CT after surgical mass removal. CT = computed tomography.

An initial physical examination revealed 4/5 motor weaknesses in the upper and lower right limbs. Cognitive function and cranial nerve evaluations were normal. The laboratory findings were nonspecific. The levels of tumor markers, carcinoembryonic antigen (CEA), and cancer antigen 19-9 were within the normal ranges. Contrast-enhanced brain computed tomography (CT) showed a mass of approximately 32 × 28 mm in the left frontal lobe with peripheral ring enhancement and vasogenic edema, indicating a primary brain tumor or metastatic tumor (Fig. 1A).

**Figure 2.**
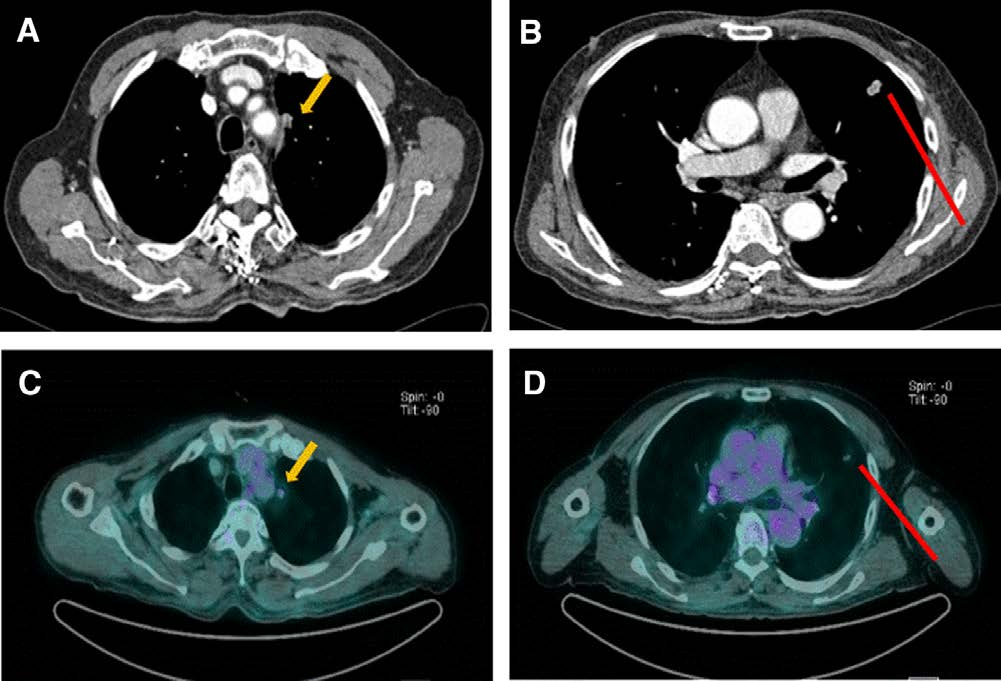
Contrast-enhanced Chest CT (A, B) and PET-CT (C, D). (A, B) Chest CT revealed 9.5 mm sized speculated nodule in left upper anterior segment (yellow arrow) and 15.2 mm sized irregular nodule in left lingular segment (red row). (C, D) PET-CT showed mild FDG-uptake in respectively. CT = computed tomography, FDG = 18-Fluoro-deoxyglucose, PET = Positron emission tomography.

The patient was subsequently admitted and administered intravenous mannitol (200 mg, q 6hr) and corticosteroid (methylprednisolone 125 mg, q 6hr) to relieve his neurological symptoms. Two days later, the tumor mass was removed under general anesthesia. Sufficient tumor tissue was obtained. After the operation, follow-up brain CT showed that almost all of the mass had been removed, and there were no postoperative complications (Fig. 1B). Simultaneously, Chest CT, abdomen–pelvic CT and 18-Fluoro-deoxyglucose positron emission tomography (FDG-PET) were performed to identify other recurrence sites. Chest CT showed a 9.5 mm sized speculated nodule in the left upper anterior segment (Fig 2A, arrow) and a 15.2 mm sized irregular nodule in the left lingular segment (Fig 2B, row), both of which exhibited heterogeneous enhancement and mild FDG-uptake on PET-CT (Fig. 2C and D). Additionally, there was no evidence of intra-abdominal recurrence of CCA.

**Figure 3.**
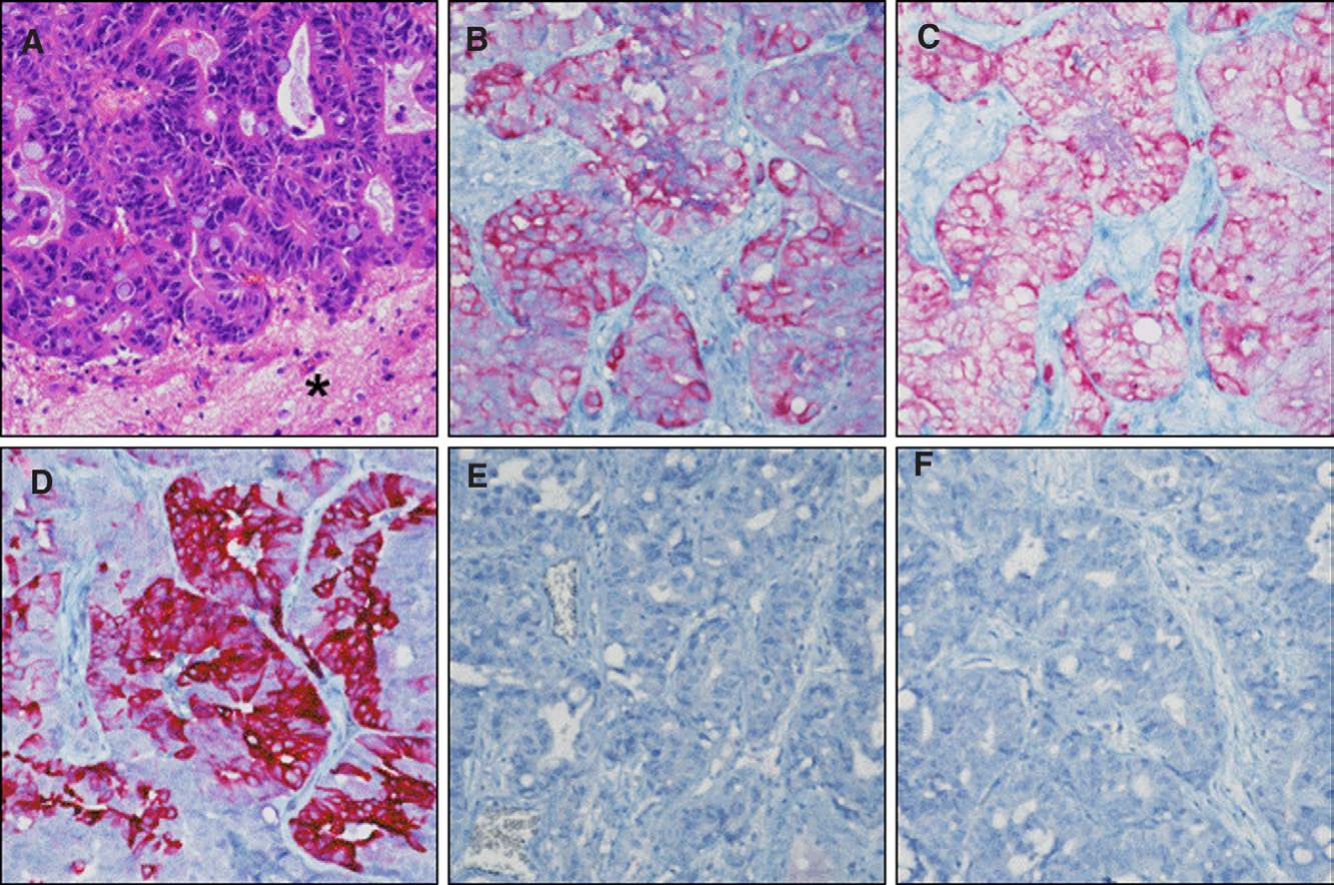
Histologic findings of metastatic cholangiocarcinoma to the brain (×400 magnification). (A) Metastatic cholangiocarcinoma in brain parenchyma (asterisk). (B–F) Tumor cells were positive for CK7 (B), CK 19 (C), and CK 20 (D), but negative for TTF-1 (E) and napsin A (F). CK = cytokeratin, TTF = thyroid transcription factor.

Pathologic examination revealed metastatic adenocarcinoma, for which immunohistochemistry (IHC) analysis was negative for thyroid transcription factor (TTF)-1 and napsin A, and positive for cytokeratin (CK) 7, CK20, and CK19 (Fig. 3).

Considering these CT, PET-CT, and pathologic findings, very late recurrence of BTC with brain and lung metastases was suggested. A therapeutic plan involving systemic chemotherapy with gemcitabine and cisplatin was proposed, but the patient refused further chemotherapy. Consequently, the patient was discharged, and the subsequent follow-up was lost.

## 3. Discussion

Our case presents a unique scenario of very late brain metastasis from CCA preceding locoregional recurrence in the liver, a phenomenon rarely documented in the literature. The absence of locoregional recurrent lesions and primary tumors in the liver and abdominal cavity posed diagnostic challenges, leading us to consider the possibility of a primary brain malignancy or a metastatic tumor from a new malignancy.

The lack of suspicious locoregional lesions and the presence of only 2 small-sized, heterogeneously enhanced nodules in the lungs raised questions about the origin of these lesions. CCA was meticulously diagnosed through a comprehensive assessment, including IHC results. The positive expression of CK7 and CK19, commonly associated with CCA, coupled with the absence of TTF-1 and napsin, indicates a distinctive immunostaining pattern.^[5]^ This intricate diagnostic process was further enriched by a tumor board discussion, in which multidisciplinary experts collectively reviewed the patient’s case, incorporating past medical records highlighting a history of CCA and subsequent brain metastasis. Ultimately, the combined consideration of pathological analysis and the documented history of previous CCA led to the conclusive determination that the origin of brain metastasis was linked to the earlier CCA.

Although next-generation sequencing (NGS) could have been utilized to identify the primary focus,^[5]^ the lack of adequate facilities within the hospital at that time precluded its implementation. Despite the limitations imposed by the constraints of the available diagnostic tools and technologies at the time, the case underscores the need for ongoing efforts to enhance the accuracy of diagnoses in such situations.

Metastatic brain tumors are the most common intracranial neoplasms in adults, affecting approximately 8% of individuals diagnosed with cancer.^[6]^ Brain metastases from BTCs are exceptionally rare, accounting for only a small percentage of intracranial neoplasms in adults, with lung, breast, and melanoma being the most common.^[7]^ The prognosis of patients with brain metastasis is typically poor, given the challenges associated with the limited penetration of traditional systemic chemotherapies through the blood-brain barrier. Retrospective analyses by Falkson et al and Frega et al reported median OS times of approximately 7 and 3 months, respectively, highlighting the complexity of treatment decisions.^[3,7]^

Supportive care remains the predominant treatment modality for brain metastasis, constituting 47% of the cohorts.^[8]^ For patients tolerating therapies, local treatments such as neurosurgical resection, stereotactic radiosurgery (SRS), and whole-brain radiation therapy (WBRT) are considered as treatment options. Surgery is highly recommended for symptomatic patients, especially those with a single lesion, as in the present case. In our case, postoperative brain CT imaging revealed successful removal of the mass (Fig. 1B), leading to the decision not to pursue additional treatment, such as WBRT.

In recent years, new immunotherapies have demonstrated efficacy and value for various cancers. Notably, durvalumab, which is a selective, high-affinity human immunoglobulin G1 monoclonal antibody that inhibits programmed cell death protein-1 (PD-L1), binds to PD-1 and CD 80, combined with gemcitabine plus cisplatin is approved as a frontline treatment for advanced BTC.^[9]^ However, owing to the low incidence of brain metastases in patients with CCA, there is a lack of clinical data on intracranial responses to durvalumab. Ongoing trials in other primary cancers, such as extensive-stage small cell lung cancer, may provide insights into its effects on brain metastases,^[10]^ potentially expanding the treatment options for CCA patients with this rare complication. In addition, Peiyi et al^[11]^ reported a complete response of metastatic brain lesions from iCCA when treated with camrelizumab (300 mg intravenously every 3 weeks) plus lenvatinib (8 mg orally once daily), supporting the use of immunotherapy in CCA with brain metastasis.

The frequency of CCA occurrence exhibits notable regional variations, with less frequent cases observed in Western nations and a higher prevalence in Asia, especially in Korea, China and Thailand.^[12]^ Notably, this case represents the first reported instance of late recurrence of brain metastasis from extrahepatic CCA in Korea. Enrolling patients with brain metastases in CCA for clinical trials remains challenging due to their extremely low incidence. However, the urgent development of targeted therapies, immune checkpoint inhibitors, and clinical studies focusing on the direct antieffect on intracranial lesions are essential to improve the prognosis of this fatal disease.

In conclusion, our case highlights the challenges of diagnosing and managing very late brain metastasis in CCA. In this case, neurologic symptoms due to brain metastasis occurred very late without involvement of liver or bones, which is very rare metastatic pattern of CCA and makes this case significant. Furthermore, while the presence of small metastases in the lung could potentially lead to a misdiagnosis of lung cancer with brain metastasis, but clinical and pathological evaluations, especially IHC was crucial in identifying the primary cancer origin.

Informed written consent was obtained from the patient for publication of this case report and accompanying images. This study was approved by the Institutional Review Board of Jeonbuk National University Hospital.

## Author contributions

**Conceptualization:** Seungah Cha, So-Yeon Jeon.

**Investigation:** Seungah Cha, Chang-Hoon Lee, Kyu Yun Jang.

**Visualization:** Seungah Cha, Chang-Hoon Lee.

**Writing – original draft:** Seungah Cha.

**Supervision:** So-Yeon Jeon.

**Writing – review & editing:** So-Yeon Jeon.
